# The Miscellaneous Medical Works of Dr. Ernst Ludwig Heim

**Published:** 1837-10

**Authors:** 


Art. III.—Dr. Ernst Ludwig Heim's *Vermischte Medicinische Schriften. Im Auftrage des Verfassers nach hinterlassenen Papieren gesammelt, und herausgegeben von* Dr. A. Paetsch, Ausübendem Arzte zu Berlin.—*Leipzig*, 1836. 8vo. pp. 412.

*The Miscellaneous Medical Works of* Dr. Ernst Ludwig Heim. *Collected and Published, at the Author's desire, from his posthumous papers, by* Dr. A. Paetsch, Medical Practitioner in Berlin.—8vo. pp. 412.

This is one of those works which serve as links to connect the present with the bygone times. The writings contained in it extend over a period of fifty years, and have, with the exception of one, been already published in the periodical publications of the day. They are evidently the fruit of a powerful, active, and discriminating mind, eminently gifted with the faculty of accurate observation. They are composed partly of original papers, and partly of reviews of the works of others. Many of them have now, owing to the late rapid progress of practical medicine, lost much of their value, and it is to be regretted that the bulk of the present volume has thus been unnecessarily swelled. To such belong several papers on the diseases of cattle, which the late progress of veterinary medicine has rendered almost worthless. There are others, such as those upon vaccination, which still possess great interest, not, however, so much in a practical, as in a philosophical point of view, by shewing the progress of vaccination through the various stages of its reception till its almost universal adoption, and thus imparting even now a personal interest, as it were, in the controversy, which, at the period of their first publication, was carried on between the promoters and adversaries of the new doctrines.

The reviews are able and well written, and valuable as indicative of the knowledge and acuteness of the writer, but from the cause already alluded to they have lost much of their pristine worth. A few of the original papers still remain unaffected by recent discovery, and as belonging to such we may mention an excellent article upon the diagnostic characters of scarlatina, roseola, and measles. The last paper of the series is now published for the first time, and is perhaps the best in the collection. It contains the results of Heim's experience in extra-uterine pregnancy during a practice of sixty years. During this period thirty-three cases came under his observation; of these sixteen were situated in the fallopian tubes, three in the ovaries, and fourteen in the abdominal cavity.

When complaints begin to be made, towards the fourth or sixth week of pregnancy, of severe intermitting pains in either hypogastric region, uncontrollable by any remedies except the most powerful narcotics, and accompanied by a very peculiar moaning cry, there is strong reason, says Heim, to suspect pregnancy of the fallopian tube. The cry of the patient is said indeed to be so characteristic, that it suffices to have heard it but once to be enabled almost with certainty to predict pregnancy of the tube upon again hearing it. These pains continue for a longer or shorter space of time, with intervals of relaxation more or less complete, till at last the patient is suddenly seized with the most agonising pains, accompanied sometimes by vomiting and purging, and symptoms which in more than one instance gave rise to suspicions of poisoning. Death speedily ensued, and was proved by dissection to be owing to the bursting of the fallopian tube, produced by pressure caused by the contained ovum, and the consequent escape of a large quantity of blood into the abdominal cavity.

So confident did Heim become of the accuracy with which he could, from the above-mentioned symptoms, distinguish pregnancy of the tubes, that had opportunities occurred to him after he had been enlightened by experience he would not have hesitated to have recommended extirpation of the affected tube, as the only means of saving life. He recommends, however, that the operation should not be performed unless the pains should continue for some time to increase in intensity, as the death of the ovum may preclude the necessity of operating. The operation will always remain extremely hazardous, and it is in vain to deny that the risk of committing error in the diagnosis is very considerable.

Among the cases which Heim records of abdominal pregnancy, is one in which some of the most eminent practitioners in Berlin agreed with him in predicating the presence of a full-timed fœtus in the abdominal cavity. Extraction was resolved upon, and Dieffenbach undertook to perform the operation; but on laying open the abdominal cavity no vestige of a fœtus was to be found. The woman fortunately recovered much to Heim's satisfaction, as he had exerted all his eloquence to induce her to submit to the operation. It were needless, after mentioning this case, again to advert to the extreme difficulty of attaining an accurate diagnosis in cases of pregnancy of the fallopian tube, which had not passed beyond the fourth or sixth week. In another case of abdominal pregnancy, in which Bruckert operated, the diagnosis was more correct, and a fine healthy child was extracted, which received the name of Macduff.

